# Current treatment strategies for postoperative intrahepatic bile duct stones in congenital biliary dilatation: a single center retrospective study

**DOI:** 10.1186/s12887-022-03759-4

**Published:** 2022-12-03

**Authors:** Atsuro Takimoto, Shigehisa Fumino, Masafumi Iguchi, Masakazu Takemoto, Shohei Takayama, Kiyokazu Kim, Mayumi Higashi, Shigeyoshi Aoi

**Affiliations:** Department of Pediatric Surgery, Kyoto Prefectural University of Medicine, 465 Kawaramachi-Hirokoji, Kamigyo-ku 602-8566 Kyoto, Japan

**Keywords:** Congenital biliary dilatation, Late complication, Ursodeoxycholic acid, Double-balloon endoscopy, Intrahepatic bile duct stone

## Abstract

**Background:**

Intrahepatic bile duct (IHBD) stones are one of the most common late complications of Roux-en-Y hepaticojejunostomy for congenital biliary dilatation (CBD). We report the current treatment strategies for IHBD stones and their outcomes in our institute.

**Methods:**

Between 1983 and 2021, 117 patients with CBD were surgically treated in our institute. Our treatment strategies included oral ursodeoxycholic acid (UDCA), double-balloon endoscopic retrograde cholangiography (DB-ERC), percutaneous cholangio-drainage (PTCD), and open surgery. A retrospective study was conducted using medical charts.

**Results:**

Postoperative IHBD stones were identified in 12 of 117 patients with CBD (10.2%). Five patients received UDCA, and small stones were successfully resolved in two cases. DB-ERC was performed eight times in five patients, but the endoscope could not reach the porta hepatis due to a long jejunal loop in two of five patients. One patient presented with severe acute pancreatitis induced by prolonged DB-ERC. PTCD was performed in three patients, two of whom finally underwent open surgery due to unsuccessful lithotomy. Open surgery was eventually performed in three patients. Lithotomy was performed in one patient; lithotomy with strictureplasty was performed in another patient. The other patient was diagnosed with intrahepatic cholelithiasis with adenocarcinoma. He underwent left lobectomy and died of carcinomatous peritonitis.

**Conclusions:**

Oral UDCA may be effective for small stones. Although DB-ERC should be considered as a first-line interventional therapy for lithotomy, it may not be feasible due to a long jejunal loop, and pancreatitis may occur. Long-term follow-up and early detection and treatment for IHBD stones may yield a good prognosis.

## Background

Congenital biliary dilatation (CBD) usually has an excellent short-term prognosis after prompt surgical treatment, which typically consists of total resection of the extrahepatic bile duct and reconstruction using Roux-en-Y hepaticojejunostomy (RYH). Recently, some studies concerning long-term follow-up have been reported, and the long-term results have gradually become clearer [1–4]. Intrahepatic bile duct (IHBD) stones are one of the most common late complications, especially at > 10 years after surgery for CBD. As reported in 2009, postoperative IHBD stones were identified in only 1 of 56 patients (1.7%) over 10 years of postoperative follow-up in our institute [5]. However, the number of patients with IHBD stones has recently increased, and severe sequelae due to IHBD stones (e.g., abdominal colic, cholangitis, and cholangiocarcinoma) become a major problem during follow-up.

In this study, we reviewed the current treatment strategies for IHBD stones and the outcomes of patients with IHBD stones in our institute.

## Methods

### Participants

With institutional board review approval, patients who underwent surgery for CBD between 1983 and 2021 were retrospectively reviewed. All patients had undergone total resection of the extrahepatic bile duct and RYH (length of the jejunal loop: 20-50 cm) in our institute. There were 117 (female, *n* = 84) patients. The median age at primary surgery was 3 years (range: 14 days to 28 years). The Todani classifications were as follows: type Ia, *n* = 43; Ic, *n* = 31; IVa, *n* = 30; non-dilatation, *n* = 6; and unknown, *n* = 7. Median follow-up period was 28 years (range 1–39 years). According to our follow-up policy, postoperative follow-up examinations were conducted at least once every 3–6 months within the first two years after surgery, and once per year thereafter unless there were any postoperative complications. Blood tests, including liver function tests and abdominal ultrasonography were routinely performed on each visit. Magnetic resonance cholangiopancreatography (MRCP) was performed within 0.5–1 years after surgery and approximately every few years thereafter, and if there were any postoperative abnormal findings including IHBD stones, MRCP was performed annually.

Data on demographics, interventional procedures, the hospital course, and outcomes were collected. Groups were compared using the Welch’s test for continuous variables and Fisher’s exact test for categorical variables. *P* values of < 0.05 were considered to indicate statistical significance.

### Treatment for IHBD stones

Our standard treatment strategies for postoperative IHBD stones included oral ursodeoxycholic acid (UDCA) for litholysis, double-balloon enteroscopic retrograde cholangiography (DB-ERC), percutaneous cholangio-drainage (PTCD), and open surgery.

When IHBD stones were found during postoperative follow-up, UDCA was administered with daily dose of 5–16 mg/kg especially for asymptomatic patients. After 2015, when DB-ERC was introduced in our institute, if IHBD stones did not dissolve or was symptomatic, DB-ERC was performed by the gastroenterologist using a short-type double-balloon endoscope under sedation. CO_2_ insufflation was used in all procedures. After the scope reached the site of hepatico-jejunal anastomosis going backwards through the site of Y anastomosis, biliary duct cannulation was attempted using a catheter. The effective length of the short-type DB enteroscope is 155–200 cm, which permits the use of nearly all of the devices required for standard ERC procedures. After making a diagnosis by cholangiography, endoscopic biliary interventions, such as balloon dilation of the anastomotic site and stone extraction using a basket catheter, were performed.

If DB-ERC is unavailable (as was the case before 2015) or failed, PTCD was performed for patients who required early biliary drainage. After puncture using a 21-gauge needle under ultrasound guiding, a 0.018-in. guidewire was inserted into the bile duct through the needle. The introducer set ensured access into the biliary system and facilitated the introduction of a 0.035-in. guidewire and 7Fr pig tail catheter.

Open surgery was performed for patients with refractory and recurrent IHBD stones or biliary malignancy. This included lithotomy, strictureplasty, and anatomical hepatectomy.

## Results

Postoperative IHBD stones were identified in 12 of 117 patients with CBD (10.2%). The median age at the diagnosis of IHBD stones was 11.5 (6–28) years, and the median duration from hepaticojejunostomy to the first diagnosis of hepatolithiasis was 10 (3–28) years. The comparison of background factors between patients with and without IHBD stones was shown in Table 1. The median age at primary surgery for CBD was significantly younger in the patients with IHBD stones (1 year [1 month to 4 years] vs. 3 years [14 days to 17 years]; *p* = 0.02). The Todani classification was type Ia in four patients and IVa in seven patients; the remaining patient had non-dilatation type. Type IVa was significantly more common in patients with IHBD stones. Patients with type Ic showed no postoperative IHBD stones; the difference was statistically significant (*p* = 0.03).

**Table 1:** Comparison of patients with and without postoperative intrahepatic bile duct stones

	IHBD stone (+)	IHBD stone (−)	
	*n* = 12	*n* = 105	*p*
Sex (F/M)	9 / 3	74 / 31	1.00
Median age at surgery for CBD (range)	1 year (1 month - 4 years)	3 years (14 days - 17 years)	0.02
Todani classification			
Ia	4	39	1.00
Ic	0	31	0.03
IVa	7	23	0.01
no dilatation	1	5	0.49
Median follow period (range)	16.5 years (5–39 years)	22 years (1–39 years)	0.47

*IHBD* Intrahepatic bile duct

*CBD* Congenital biliary dilatation

**Fig. 1:**
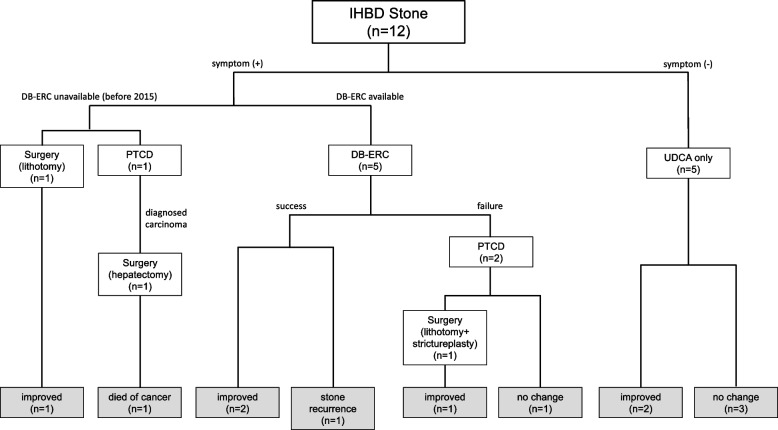
Flow chart of treatment for postoperative intrahepatic bile duct stones

Figure 1 shows the treatment course of these patients. The details of the clinical course of each patient are listed in Table 2.

**Table 2:** Summary of 12 patients with postoperative intrahepatic bile duct stones

No.	Age at surgery for CBD	Sex	Todani	Site of stenosis	Age at diagnosis of IHBD stone	Symptom due to IHBD stone	Treatment for IHBD stone	Outcome
1	1 month	F	Ia	posterior segmental branch	28 years	recurrent cholangitis abdominal pain	DB-ERC - > PTCD	no change
2	2 years,8 months	F	IVa	anastomotic stenosis	22 years	abdominal pain	DB-ERC - > PTCD - > Surgery (lithotomy+strictureplasty)	improved
3	2 years, 6 months	F	IVa	right hepatic duct	21 years	abdominal pain	DB-ERC	stone recurrence
4	3 years,3 months	F	Ia	–	10 years	abdominal pain	DB-ERC	improved
5	2 years, 8 months	F	no dilatation	anastomotic stenosis	10 years	abdominal pain	DB-ERC	improved
6	1 year, 2 months	M	Ia	–	11 years	–	UDCA only	no change
7	1 year, 7 months	F	IVa	–	8 years	–	UDCA only	improved
8	1 year, 11 months	M	IVa	–	6 years	–	UDCA only	no change
9	5 months	F	Ia	–	7 years	–	UDCA only	improved
10	4 years, 5 months	F	IVa	–	3 years	–	UDCA only	no change
11	1 year, 11 months	F	IVa	–	11 years	abdominal pain	Surgery (lithotomy)	improved
12	5 months	M	IVa	lateral segmental branch	26 years	cholangitis abdominal pain	PTCD - > Surgery (hepatectomy)	died of cancer

*CBD* congenital biliary dilatation; *IHBD* intrahepatic bile duct; *DB-ERC *double balloon endoscopic retrograde cholangiography; *PTCD* percutaneous transhepatic cholangio drainage; *UDCA *ursodeoxycholic acid

**Fig. 2:**
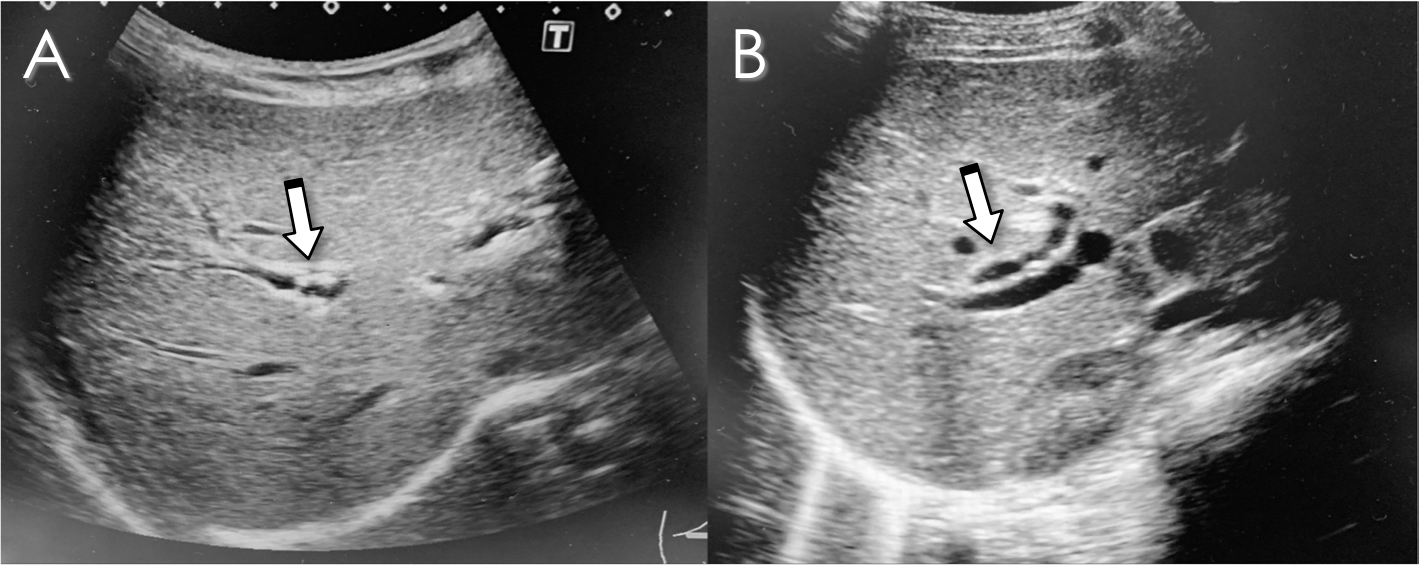
Ultrasound findings in a case in which an IHBD stone disappeared with oral UDCA. **A** Before treatment. Arrow indicates an IHBD stone in the right hepatic duct. **B** After treatment. Arrow shows the absence of the stone

Among 12 patients, five asymptomatic patients were treated by UDCA only. The median age at the diagnosis of IHBD stones was 8 years (6–11), and the median duration from primary surgery to the diagnosis was 7 years (3–10). Small stones (< 7 mm) were successfully resolved on abdominal ultrasonography at one year after the administration of UDCA in 2 cases (Fig. 2). Three of the five remained unchanged and asymptomatic, and continued taking UDCA and attending follow-up visits.

**Fig. 3:**
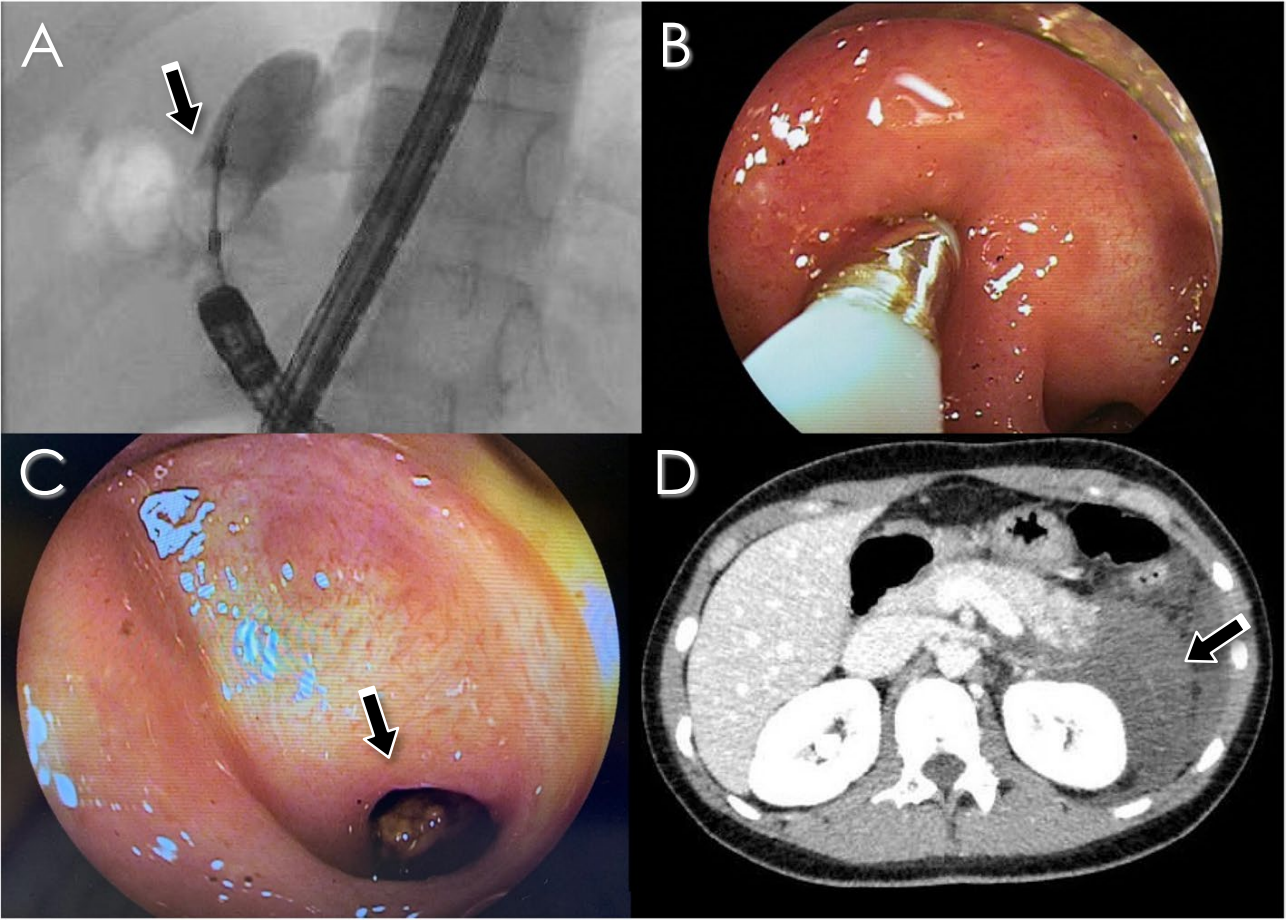
DB-ERC findings and acute pancreatitis after intervention (Case 3). **A** Cholangiography and balloon dilatation of right hepatic duct stricture. **B** In DB enteroscopy, the stone was crushed using a crusher catheter. **C** Arrow shows the IHBD stone. **D** Enhanced abdominal CT shows decreased enhancement and swelling of the pancreatic tail 1 day after DB-ERC

DB-ERC was attempted 8 times in 5 patients with hepatolithiasis. The median age of these patients at the time of primary surgery for CBD was 2 (0–3) years, and the average age at the first time of DB-ERC was 20 (9–36) years. The median duration from primary surgery to DB-ERC was 18 (8–36) years. There were no cases of bile duct plasty at primary surgery for CBD. In 2 of the 5 patients, the endoscope could not reach the porta hepatis due to a long jejunal loop and intestinal adhesion. One patient (Case 3) presented with severe acute pancreatitis induced by prolonged DB-ERC over 3 hours (Fig. 3). Although she fully recovered from pancreatitis 2 months after DB-ERC, asymptomatic IHBD stone recurrence was noted after 2 years, and the administration of UDCA was continued. The remaining two patients successfully improved.

PTCD for the removal of the stone was attempted in 3 patients after primary surgery for CBD. Case 1 presented recurrent cholangitis with dilatation of the posterior segmental branch. After an unsuccessful attempt at DB-ERC, PTCD was attempted; however, there was a risk of vascular injury, and puncture was unsuccessful. In Case 2, DB-ERC was attempted but failed and subsequent PTCD for establishing internal drainage and lithotomy using percutaneous balloon catheter via the route were successful. However, balloon dilation didn’t improve anastomotic stenosis and internal bile drainage was required. Finally, open surgery including lithotomy with strictureplasty of hepatico-jenunal anastomosis was performed due to recurrence of stones and repeated drainage tube occlusion 5 years after PTCD. As previously reported, Case 12 (before 2015) presented multiple stones and stenosis of the lateral segmental branches of the intrahepatic bile duct. PTCD was performed and histological examination of a biopsy specimen obtained from the left hepatic duct revealed adenocarcinoma. He underwent left lobectomy and died of carcinomatous peritonitis one year after hepatectomy [6]. Lithotomy via the anterior wall opening of the jejunal loop was performed in Case 11.

Finally, 6 of 12 patients with IHBD stones improved. The remaining five patients continue to receive UDCA and outpatient follow-up.

## Discussion

IHBD stones are the most common late complications after radical surgery for CBD, with an incidence rate of 2.7–11% [7]. It is hypothesized that postoperative bile stasis and infection of the biliary tract cause the formation of IHBD stones. The main causes of these conditions are anastomotic stricture, intrahepatic bile duct stricture, and residual intrahepatic ductal dilatation [2]. Therefore, the formation of hepatic stones after definitive surgery is more common in type IVa, as this study presented. Primary stricture of the hepatic ducts near hilum and intrahepatic ducts is occasionally seen in patients with CBD, and whenever possible these ducts should be widened by duct plasty at the time of the primary surgery [1]. In this study, 3 type Ia cases without apparent stenosis showed postoperative IHBD stones, and we speculated that the suture remnant might become the core of such stones. Postoperative stenosis and repeated cholangitis of the intrahepatic bile duct lead to bile stasis and IHBD stones, and may be associated with a high risk of carcinogenesis [4]. The incidence of biliary carcinoma among patients who undergo radical surgery is reported to be approximately 0.7–5.4%, which is higher than that in the general population [4]. Therefore, symptomatic IHBD stones, which may promote these conditions should be considered for treatment.

Various treatments have been reported for IHBD stones after resection of CBD, including UDCA, DB-ERC, hepatectomy, revision of hepatico-enterostomy, and extracorporeal shockwave lithotripsy [8, 9]. The surgical approach is still challenging because it requires wide laparotomy, the release of intestinal adhesion, division of bilioenteric anastomosis, removal of stones from the intrahepatic bile duct, and re-anastomosis of the hepaticojejunostomy. With the development of endoscopic technology in recent years, DB-ERC has been widely available, even in the pediatric population [10, 11]. In our institute, DB-ERC was introduced from 2015, and we applied DB-ERC as a first-line therapeutic method for pediatric patients with IHBD stones after RYH. DB enteroscopy has made it possible to reach the site of hepatico-jejunal anastomosis endoscopically via the jejunal loop of the RYH, and to successfully perform therapeutic interventions and removal of IHBD stones using a crush catheter or basket catheter. The success rate of DB-ERC in pediatric cases is reported as 76.9% [11]. In our series, the endoscope could not reach the porta hepatis because of intestinal adhesion and a long jejunal loop in 2 of 5 patients. Therefore, physicians need to take care of the length of the jejunal loop in the primary surgery. DB-ERC has been associated with a higher risk of adverse events when applied to patients with surgically altered gastrointestinal anatomy. The incidence of DB-ERC-related adverse events has been reported to be 5.4–10.6% [11, 12]. In this study, acute pancreatitis was observed as an adverse event of DB-ERC. The causal mechanism of post-DBE acute pancreatitis is uncertain. Possible mechanisms are as follows: direct trauma of the pancreas caused by pressure of the endoscope against the vertebral column; disorder of microcirculation during the procedure; increased intraluminal duodenal pressure during the endoscopic procedure; and reflux of duodenal fluids into the pancreatic duct leading to acute pancreatitis [13]. The incidence of residual and recurrent stones after DB-ERC with stone extraction was higher than after percutaneous transhepatic cholangioscopy and surgery [14]. Physicians should be aware of these complications related to DB-ERC. Recently, new treatments for IHBD stones such as percutaneous transhepatic endoscopic biliary holmium laser lithotripsy and endoscopic ultrasound-guided-hepaticogastrostomy have been reported [15, 16]. Complicated or large biliary IHBD stones could be treated successfully using these treatments, if a pathophysiology and an anatomical conditions match. Although these novel treatments have not been implemented in our institute yet, these would be promising alternative methods for refractory cases.

UDCA is currently employed for oral litholysis of small cholesterol gallstones. The proportion of this bile acid is increased in the bile acid pool, inducing decreased hepatic secretion of biliary cholesterol and the formation of unsaturated bile, the key factor that promotes the dissolution of cholesterol crystals [17]. UDCA is reported to have a role in reducing the size of gallstones in pediatric patients [18]. In this study, small stones disappeared in 2 patients with hepatolithiasis who received oral UDCA. As long as the patients are asymptomatic, it is important to prescribe UDCA and do active surveillance using ultrasonography or MRCP. If IHBD stones become symptomatic or cause biliary obstruction, the intervention including DB-ERC, PTCD would be considered.

## Conclusions

Oral UDCA may be effective for small stones. Although DB-ERC should be considered as a first-line interventional therapy for lithotomy, it may be infeasible due to a long jejunal loop, and pancreatitis may occur. Long-term follow-up and early detection and treatment of IHBD stones may provide a good prognosis.

## Data Availability

All data from this study are not publicly available due to compliance to privacy. Summaries are available from the corresponding author on reasonable request.

## Abbreviations

IHBD: Intrahepatic bile duct
CBD: Congenital biliary dilatation
RYH: Roux-en-Y hepaticojejunostomy
UDCA: Ursodeoxycholic acid
DB-ERC: Double-balloon endoscopic retrograde cholangiography
MRCP: Magnetic resonance cholangiopancreatography
PTCD: Percutaneous cholangio-drainage

## References

[1] Todani T, Watanabe Y, Urushihara N, Noda N, Morotomi Y (1995). Biliary complications after excisional procedure for Choledochal cyst. J Pediatr Surg.

[2] Urushihara N, Fukumoto K, Fukuzawa H, Mitsunaga M, Watanabe K, Aoba T, Yamoto M, Miyake H (2012). Long-term outcomes after excision of choledochal cysts in a single institution: operative procedures and late complications. J Pediatr Surg.

[3] Mukai M, Kaji T, Masuya R, Yamada K, Sugita K, Moriguchi T, Onishi S, Yamada W, Kawano T, Machigashira S, Nakame K, Takamatsu H, Ieiri S (2018). Long-term outcomes of surgery for choledochal cysts: a single institution study focusing on follow-up and late complications. Surg Today.

[4] Amano H, Shirota C, Tainaka T, Sumida W, Yokota K, Makita S, Takimoto A, Tanaka Y, Hinoki A, Kawashima H, Uchida H (2021). Late postoperative complications of congenital biliary dilatation in pediatric patients: a single center experience of managing complications for over 20 years. Surg Today.

[5] Ono S, Fumino S, Shimadera S, Iwai N (2010). Long-term outcomes after hepaticojejunostomy for choledochal cyst: a 10- to 27- year follow-up. J Pediatr Surg.

[6] Ono S, Sakai K, Kimura O, Iwai N (2008). Development of bile duct cancer in a 26-year-old man after resection of infantile choledochal cyst. J Pediatr Surg.

[7] Shirota C, Kawashima H, Tainaka T, Sumida W, Yokota K, Makita S, Amano H, Takimoto A, Hinoki A, Uchida H (2021). Double-balloon endoscopic retrograde cholangiography can make a reliable diagnosis and good prognosis for postoperative complications of congenital biliary dilatation. Sci Rep.

[8] Clemente G, Giuliante F, De Rose A.M., Ardito F, Nuzzo Get. Liver resection for intrahepatic stones in congenital bile duct dilatation. J Visc Surg 2010;147:(3)175–180. 10.1016/j.jviscsurg.2010.06.005 20709617

[9] Okada Y, Miyamoto M, Yamazaki T, Motoi I, Kuribayashi M, Kodama K (2007). Piezoelectric extracorporeal shockwave lithotripsy for bile duct stone formation after choledochal cyst excision. Pediatr Surg Int.

[10] Ono S, Maeda K, Baba K, Usui Y, Tsuji Y, Yano T, Hatanaka W, Yamamoto H (2013). The efficacy of double-balloon enteroscopy for intrahepatic bile duct stones after roux-en-Y hepaticojejunostomy for choledochal cysts. Pediatr Surg Int.

[11] Yokoyama K, Yano T, Kumagai H, Mizuta K, Ono S, Imagawa T, Yamamoto H, Yamagata T (2016). Double-balloon Enteroscopy for pediatric patients evaluation of safety and efficacy in 257 cases. J Pediatr Gastroenterol Nutr.

[12] Shimatani M, Hatanaka H, Kogure H, Tsutsumi K, Kawashima H, Hanada K, Matsuda T, Fujita T, Takaoka M, Yano T, Yamada A, Kato H, Okazaki K, Yamamoto H, Ishikawa H, Sugano K (2016). Diagnostic and therapeutic endoscopic retrograde cholangiography using a short-type double-balloon endoscope in patients with altered gastrointestinal anatomy: a multicenter prospective study in Japan. Am J Gatroenterol.

[13] Kopáčová M, Bureš J, Rejchrt S, Vávrová J, Bártová J, Soukup T, Tomš J, Tachecí I (2016). Risk factors of acute pancreatitis in Oral double balloon Enteroscopy. Acta Med (Hradec Kralove).

[14] Suzuki Y, Mori T, Yokoyama M, Nakazato T, Abe N, Nakamura Y, Tsubouchi H, Sugiyama M (2014). Hepatolithiasis: analysis of Japanese nationwide surveys over a period of 40 years. J Hepatob Pancreat Sci.

[15] Anna I, Federico F, Mario P, Chiara F, Eugenio C, Sergio S, Hatem A, Alberto M, Gianpaolo C, Renzo D (2013). Percutaneous transhepatic endoscopic holmium laser lithotripsy for intrahepatic and choledochal biliary stones. Int J Surg.

[16] Ishii S, Koga H, Saito H, Seo S, Ushio M, Takahashi S, et al. Endoscopic ultrasound-guided hepaticogastrostomy in a seven-years-old girl. Intern Med. 2022. 10.2169/internalmedicine.9355-22. 10.2169/internalmedicine.9355-22 PMC9790776 35491132

[17] Portincasa P, Ciaula A, Bonfrate L, Wang D (2012). Therapy of gallstone disease: what it was, what it is, what it will be. World J Gastrointest Pharmacol Ther.

[18] Weng SC, Lee HC, Yeung CY, Chan WT, Liu HC, Jiang CB (2020). Choledochal cyst as an important risk factor for pediatric gallstones in low-incidence populations: a single-center review. Pediatr Neonatol.

